# The Role of Tourniquet Use in Arthroscopic Meniscectomy: A Systematic Review

**DOI:** 10.3390/jcm15052086

**Published:** 2026-03-09

**Authors:** Cosmin Ioan Faur, Dennis Cicio, Andrea Pasquini, Edna Iordache, Jenel Marian Patrascu, Jenel Marian Patrascu, Alessandro Iatarola, Horea Benea, Octav Russu, Vlad Predescu

**Affiliations:** 1 Department of Orthopedics and Traumatology, “Victor Babes” University of Medicine and Pharmacy, 300041 Timisoara, Romania; faur.cosmin@umft.ro (C.I.F.);; 2 Orthopedics II Research Center, “Pius Brinzeu” Emergency Clinical County Hospital, 300723 Timisoara, Romania; 3 Research Center University “Professor Doctor Teodor Șora”, Victor Babes University of Medicine and Pharmacy, 300041 Timisoara, Romania; 4 Faculty of Medicine, Iuliu Hatieganu University of Medicine and Pharmacy, 400347 Cluj Napoca, Romania; cicio.dennis@elearn.umfcluj.ro (D.C.); benea.horea@umfcluj.ro (H.B.); 5 Department of Orthopedics and Traumatology, “Pius Brinzeu” Emergency Clinical County Hospital, 300723 Timisoara, Romania; andrea.pasquini@umft.ro (A.P.); alessandro.iatarola@umft.ro (A.I.); 6 Center for Modeling Biological Systems and Data Analysis, Victor Babes University of Medicine and Pharmacy, 300041 Timisoara, Romania; 7 Doctoral School of Medicine, “Victor Babes” University of Medicine and Pharmacy, 300041 Timisoara, Romania; 8 Artro Sport Clinic, 020513 Bucharest, Romania; ednaiordache1@gmail.com; 9 Department of Orthopedics and Traumatology I, George Emil Palade University of Medicine, Pharmacy, Science and Technology of Targu Mures, 540139 Targu Mures, Romania; octav.russu@umfst.ro; 10 Orthopedics and Traumatology Department, Ponderas Academic Hospital, 014142 Bucharest, Romania; vlad.predescu@reginamaria.ro

**Keywords:** arthroscopic partial meniscectomy, knee arthroscopy, meniscus tear, postoperative recovery, surgical outcomes, tourniquet

## Abstract

**Background and Objectives:** The role of tourniquet use in arthroscopic partial meniscectomy remains debatable. While traditionally adopted to enhance visualization and reduce intraoperative bleeding, concerns were raised regarding its impact on postoperative outcomes and potential adverse effects, such as muscle damage or delayed recovery. This systematic review aimed to evaluate whether the use of a tourniquet offers advantages in terms of surgical efficiency, patient recovery and complication rates in arthroscopic partial meniscectomy. **Materials and Methods:** A systematic review was conducted following PRISMA guidelines and registered in the PROSPERO database (CRD42025644740). A comprehensive literature search was performed in 5 databases including studies from the past 20 years. Only randomized controlled trials (RCTs) comparing tourniquet-assisted versus non-tourniquet procedures in adolescent and adult patients undergoing isolated arthroscopic partial meniscectomy matched our inclusion criteria and the analysis was performed on those. Methodological quality was assessed using the Cochrane RoB 2.0 tool. Data were synthesized either quantitatively or narratively, depending on the availability of statistical details. **Results:** Three RCTs with a total of 243 patients met the inclusion criteria. Operative time was shorter in tourniquet-assisted procedures in one study (*p* = 0.001), though comparable outcomes were achieved in non-tourniquet groups when pharmacological agents such as intra-articular adrenaline were used. No significant differences were observed between groups regarding postoperative pain (*p* = 0.22, *p* = 0.43), knee effusion (*p* = 0.96), range of motion (*p* = 0.91, *p* = 0.96), or time to return to functional activities (*p* = 0.9, *p* = 0.34, *p* = 0.23). Muscle damage, assessed by serum creatine phosphokinase CPK levels, did not differ between groups (*p* = 0.3, *p* = 0.093, *p* = 0.079). Intraoperative visibility and surgeon satisfaction rated higher in tourniquet groups (*p* = 0.002), although this was subjective and reported variably. No major tourniquet-related complications were recorded. **Conclusions:** The routine use of a tourniquet in arthroscopic partial meniscectomy provides limited intraoperative advantages and does not improve postoperative outcomes. Current evidence supports a selective rather than routine use of tourniquets, especially when pharmacological alternatives are available. Further high-quality studies are needed to define standardized protocols and assess long-term outcomes.

## 1. Introduction

Arthroscopic meniscectomy is a minimally invasive orthopedic procedure that is widely performed due to its efficacy in managing symptomatic meniscal injuries [1]. Surgical technique and technology have advanced to the point where this procedure means rapid recovery with very low morbidity [2,3,4]. Still, the clinical significance of using a tourniquet in arthroscopic meniscectomy is debatable.

In arthroscopic procedures, the standard use of the pneumatic tourniquet has been to create a bloodless surgical field [5,6]. While some studies report superior intraoperative visualization and reduced operative time with tourniquet use, others find no substantial difference in these outcomes, instead highlighting potential adverse effects that typically develop after 30 to 60 min of cuff inflation, including increased pain and muscle damage. Other reported complications include reperfusion syndrome, nerve injuries, skin abrasion, thigh postoperative pain, compartment syndrome, and delayed recovery of muscle function [7,8,9,10,11,12,13,14,15]. Many surgeons now question whether it is really necessary to use a tourniquet for every limb procedure, especially since newer methods are available, such as improved irrigation systems and pharmacologic agents like epinephrine and ropivacaine [2,16].

Recent meta-analyses and randomized controlled trials have added to this uncertainty, yielding mixed results on important outcomes like postoperative pain, rehabilitation rates, long-term muscle function and the much-feared venous thromboembolism [8,17,18,19,20,21,22,23,24]. Given the conflicting evidence, a comprehensive and systematic evaluation of the available literature is warranted to clarify the role of tourniquet use in arthroscopic meniscectomy.

This review aimed to compare outcomes from tourniquet-assisted procedures and non-tourniquet procedures in three areas: surgical efficiency, postoperative recovery, and complication rates.

## 2. Materials and Methods

### 2.1. Study Design, Protocol and Registration

This systematic review was designed and conducted following the recommendations of the Cochrane Handbook for Systematic Reviews of Interventions to ensure its quality and reliability [25]. It was reported using the Preferred Reporting Items for Systematic Reviews and Meta-Analyses (PRISMA) guidelines [26], and the PRISMA 2020 for Abstract Checklist and PRISMA 2020 Checklist are provided in the Supplementary Materials (Supplementary Materials S1 and S2). Due to the heterogeneity of the included studies, a pooled meta-analysis was not feasible; thus, the results were synthesized narratively in the form of a systematic review. The study protocol was prospectively registered in the PROSPERO database (ID: CRD42025644740) [27]. Since the review was based exclusively on published data, no ethical approval was required.

### 2.2. Information Sources and Literature Search Strategy

A systematic literature search was conducted using the following bibliographic databases: Cochrane Library, U.S. National Library of Medicine (PubMed/MEDLINE), Web of Science, Scopus and Google Scholar, with filters applied to include records published within the past 20 years in order to capture modern arthroscopic techniques and anesthesia protocols.

The research question was formulated using the PICO framework (Table 1).

A systematic search was performed by two independent authors (A.P. and D.C.) on 3 January 2025, integrating the following search terms: “meniscectomy”, “arthroscopic meniscectomy”, “tourniquet” and “no tourniquet”. The references cited in the identified studies were also examined to uncover any additional studies that might meet the inclusion criteria. A full detailed search strategy is available in Appendix A.

### 2.3. Inclusion and Exclusion Criteria

Inclusion criteria were: (I) adolescent and adult patients undergoing arthroscopic partial meniscectomy for symptomatic meniscal tears, (II) studies evaluating outcomes in arthroscopic meniscectomy with or without tourniquet use, (III) levels I to IV of evidence: randomized controlled trials (RCTs), controlled (non-randomized) clinical trials (CCTs), prospective and retrospective comparative cohort studies, case–control studies and case series, (IV) human subjects, (V) articles written in English and (VI) full text available.

Exclusion criteria were: (I) studies with other concomitant procedures besides arthroscopic meniscectomy, (II) articles that do not contain original patient data: reviews, editorial, commentary, letter; (III) case reports, and (IV) animal studies, in vitro/in vivo studies.

#### Participants

Adolescent and adult patients who had a meniscal tear diagnosed and underwent isolated partial arthroscopic meniscectomy (with or without tourniquet use) were studied.

To eliminate potential confounding effects on outcomes, patients undergoing concomitant procedures, such as anterior cruciate ligament (ACL) reconstruction, were excluded.

When available, demographic data, such as age, sex and comorbidity profiles, were extracted to characterize the study cohorts and assess potential baseline imbalances.

### 2.4. Study Selection Process, Data Extraction and Data Analysis

Article selection was performed by two authors (D.C. and A.P.) in a blinded way, to eliminate inter-observer bias (Appendix B). Disagreements were resolved by a third author (C.F.). Data extraction was independently performed by two authors (D.C. and A.P.) using a spreadsheet in Microsoft Excel, version 16.0 (Microsoft Corporation, Redmond, WA, USA). Predefined data were extracted: (I) study information (author, year of publication and study design), (II) patient demographics, and (III) main outcomes, as mentioned in the PICO framework (Table 1). All extracted data underwent cross-verification, with disagreements resolved by consensus.

### 2.5. Quality Assessment and Risk of Bias

Two authors (D.C. and E.I.) independently performed the quality assessment for each included study, using the scoring protocol developed by the Oxford Centre for Evidence-Based Medicine to determine levels of evidence (LoE) [28]. Assessors were not blinded to the authors of the publications.

Cochrane Risk of Bias tool (RoB 2.0) was used for qualitative assessment [29]. Findings regarding the level of evidence and risk of bias were subsequently compared and any disagreements were resolved by a senior reviewer (V.P.).

### 2.6. Outcome Measures

The outcome measures were identified through study screening. Based on their direct impact on patient recovery and clinical relevance, they were categorized as primary or secondary (Table 2).

Each outcome was extracted as reported in the original studies. When applicable, data were synthesized either quantitatively or narratively, depending on the availability of statistical details.

## 3. Results

### 3.1. Study Selection

**Figure 1:**
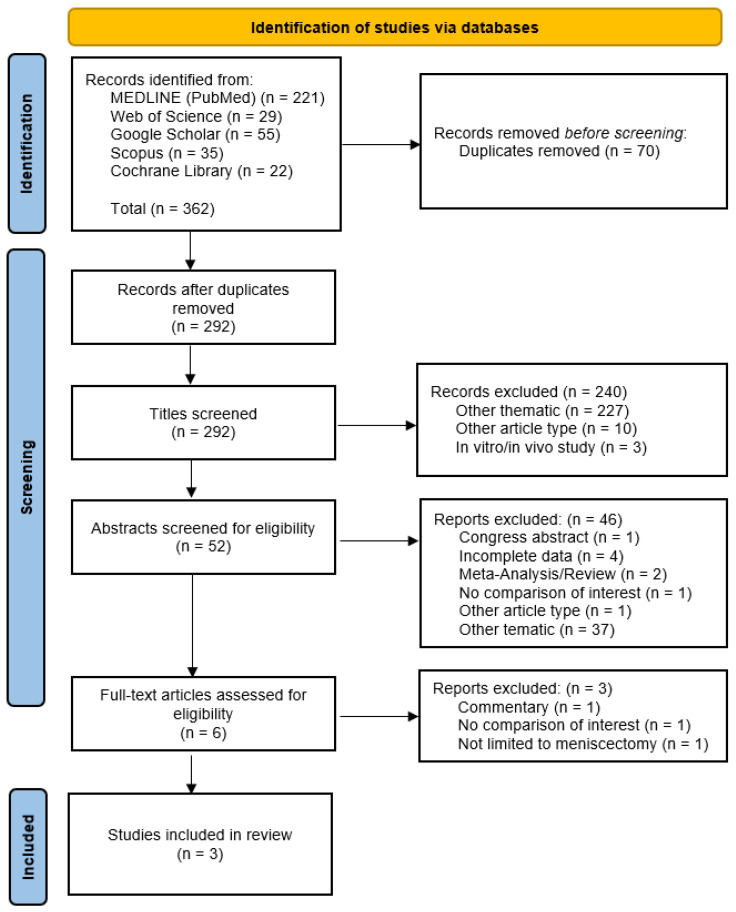
Flowchart of the literature search.

Literature search initially retrieved 362 articles identified in five databases, of which 70 were duplicates. The abstracts of 52 remaining studies were screened against inclusion and exclusion criteria. According to the eligibility criteria, three RCTs were included in the review, with a total of 243 patients [2,3,30]. No additional studies were found during the cross-referencing process. The PRISMA flowchart summarizing the study selection process is provided in Figure 1.

### 3.2. Study Characteristics

All three included studies were RCTs, classified as level IB evidence according to the Oxford Centre for Evidence-Based Medicine and published between 2010 and 2021 [28]. Total sample size was 243, with individual studies ranging from 60 to 103 subjects. Participant age ranged from 15 to 81 years, with approximately 60% being male. All procedures were partial arthroscopic meniscectomies and tourniquet protocols showed slight variations across the studies, with inflation pressures ranging from 320 to 350 mmHg. In one study, the pressure was set 100–150 mmHg above the systolic blood pressure. In all studies, the tourniquet was applied at the thigh level. Control groups varied in methodology: in some cases, pharmacological agents such as adrenaline were used to improve visualization in the absence of a tourniquet. Details of individual studies, including intervention protocols, are summarized in Table 3.

### 3.3. Quality Assessment and Risk of Bias of the Included Studies

**Figure 2:**
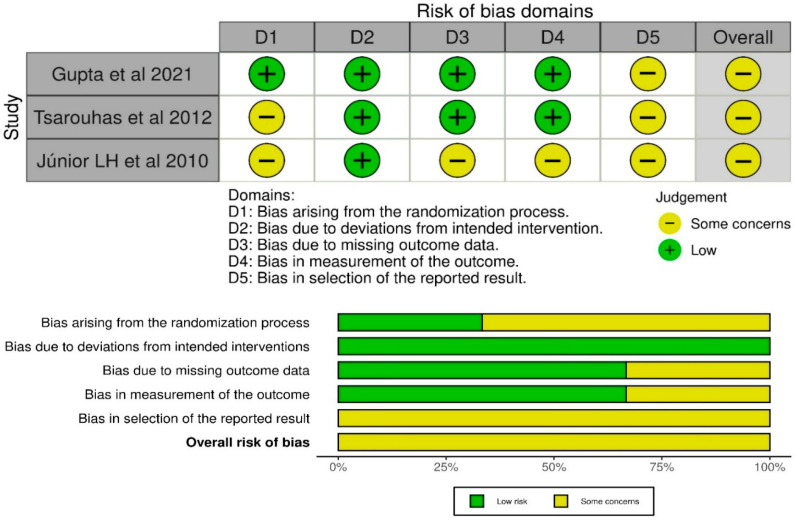
RoB2 quality assessment of included studies [2,3,30].

Methodological quality of included studies was assessed using the Cochrane RoB 2.0 tool. One study scored 9/10, indicating low risk of bias, one scored 8/10 (moderate risk), and one scored 6/10 (high risk of bias), primarily due to concerns related to the randomization process and the selection of reported outcomes (Figure 2).

### 3.4. Result of Individual Studies

This section presents the findings from the three randomized controlled trials included in this systematic review (Table 4). Evaluated outcomes comprised intraoperative efficiency, postoperative recovery, and biochemical as well as subjective measures. Due to variability in study designs and outcome reporting, a qualitative synthesis was performed. Studies included in this review evaluated a range of intraoperative and postoperative outcomes using various standardized measures. The following section presents findings with corresponding rationale for each outcome, including quantification method.

One primary parameter assessed was operative time. This measure reflects how efficient the procedure was and indirectly indicates the intraoperative difficulty and visualization quality. In the study by Gupta et al., the average operative time in the tourniquet (T) group was significantly shorter at 19.25 min compared to 26.25 min in the non-tourniquet (NT) group without adrenaline (*p* = 0.001). Operative time decreased interestingly to 20.75 min when adrenaline was introduced (non-tourniquet group, NT2), making it comparable to tourniquet-assisted procedures. In contrast, Tsarouhas et al. reported no significant differences between T and NT groups (27.5 ± 12.5 vs. 31.2 ± 12.1 min, *p* = 0.83). In the trial by Junior et al., operative times were nearly identical across groups, averaging around 21.29 min for the T arm and 21.71 min for the NT arm (*p* = 0.85). The study by Tsarouhas et al. evaluated postoperative range of motion (ROM), a key functional parameter, at 1 and 2 weeks after surgery. Pain, assessed with the Visual Analogue Scale at the same postoperative intervals, was also found to show no statistically significant differences between groups. Junior et al. measured knee effusion as an indicator of intra-articular inflammation. They reported similar values between tourniquet group (0.269 cm) and non-tourniquet group (0.279 cm), with no significant difference (*p* = 0.9669). Tsarouhas et al. also measured serum creatine phosphokinase (CPK) levels to evaluate muscle damage. CPK was tested before the operation and at 24 h, 8 days, and 15 days postoperatively. No significant differences were observed between groups at any time point. Intraoperative visibility and surgeon satisfaction were assessed in Gupta’s study using non-standardized visual or Likert scales at the end of the procedure. The scores were significantly higher in the tourniquet group, whereas comparable scores were seen in the non-tourniquet subgroup that received intra-articular adrenaline. Irrigation fluid used in surgery was also recorded by Gupta et al. The tourniquet group required significantly less fluid during the operation than the NT and NT2 groups. Additionally, tourniquet use was necessitated in two cases that were originally assigned to the NT group.

The randomized controlled trials included used different anesthesia protocols, which may have influenced perioperative parameters such as pain perception, muscle relaxation, and hemodynamic stability during arthroscopic partial meniscectomy. Tsarouhas et al. performed all of their procedures using combined femoral and sciatic nerve block. This regional anesthesia technique provides effective sensory and motor blockade of the knee and surrounding structures, and is commonly associated with reduced postoperative pain and opioid requirements [31]. Junior et al. performed their surgeries under neuraxial anesthesia, either using an epidural or spinal block. All the patients were positioned in supine position. This method of anesthesia allows for a profound sensory blockade below the waist and is commonly used orthopedic procedures of the lower limb. It also has the potential to influence intraoperative hemodynamics and postoperative mobilization. Gupta et al. conducted all of their procedures under general anesthesia. At the same time, local infiltration with 2% lidocaine was performed at the arthroscopic portal sites to enhance local analgesia.

Finally, time to return to function was analyzed by Tsarouhas et al., recording the number of days until patients resumed key activities. These outcomes were not significantly different between groups. All patients generally resumed weight-bearing within 8 to 21 days, returned to light work within day 15 to 36, and began jogging as early as day 24.

## 4. Discussion

This systematic review highlights the current evidence on the use of a tourniquet in arthroscopic partial meniscectomy. It analyzes three randomized controlled trials that compared tourniquet-assisted procedures to non-tourniquet techniques, with or without the use of pharmacological adjuncts. These trials help shed light on the intraoperative effects of applying a tourniquet, particularly concerning the impacts on operative time, surgical field visibility, and irrigation fluid usage. They also help clarify its impact, if any, on key postoperative parameters like pain, joint effusion, range of motion, and early functional recovery. No major tourniquet-related complications were observed in the included trials. The overall safety profile seems acceptable in the context of short-duration arthroscopic procedures.

The use of a tourniquet has traditionally been justified by its ability to reduce intraoperative bleeding and enhance visualization, thereby facilitating faster and more efficient surgical procedures [3,32,33,34]. This review supports that notion only in part. Gupta et al. demonstrated a statistically significant reduction in operative time when a tourniquet was used compared to a standard non-tourniquet approach [3]. However, this advantage disappeared when adrenaline, a vasoconstrictive agent, was introduced in the non-tourniquet group, suggesting that the hemostatic environment required for efficient arthroscopy can be recreated pharmacologically without the need for mechanical limb occlusion [2]. Additionally, the need for two patients in the non-tourniquet group to convert to tourniquet use without pharmacologic aid highlights a practical point: even if tourniquets are not universally required, they must still be readily available for those select instances in which compromised visibility necessitates a reliable means of hemostasis. This idea is reinforced by the findings related to irrigation fluid consumption. In tourniquet-assisted procedures, less fluid is used, which may mean less distension in the joints and reduced risk of postoperative complications like fluid overload or hypothermia. Although tourniquet use may offer minor advantages during surgery, its effect on recovery after the procedure seems minimal. In the randomized controlled trial conducted by Tsarouhas and colleagues, the only included study that directly evaluated postoperative pain, no meaningful differences were found between patients who received a tourniquet and those who did not. This finding suggests that using a tourniquet has little impact on postoperative pain, a key factor influencing patient comfort and early mobility [30]. These findings are consistent with previous literature, including the editorial by Williams in The Journal of Bone and Joint Surgery, American Volume, which supports the results of Tsarouhas et al. and emphasizes that brief tourniquet use (<30 min) does not negatively impact postoperative recovery [35]. This is particularly relevant in arthroscopic procedures, where soft-tissue trauma is minimal and the clinical benefits of avoiding a tourniquet may be marginal. Range of motion and knee effusion, objective markers of joint recovery, were also unaffected by tourniquet use, further supporting the hypothesis that the short-term inflammatory and mechanical effects of tourniquets are clinically minimal in this setting. These findings are in line with previous arthroscopic outcome literature on knee pathologies, such as plica resection, which also demonstrated favorable long-term outcomes [36]. Importantly, the studies reviewed did not report increased risk of delayed mobilization or impaired rehabilitation in the absence of a tourniquet. Similar results have been reported in total knee arthroplasty (TKA), where no clinically relevant differences in postoperative pain, range of motion, or length of stay were found between patients operated on with or without a tourniquet [37]. Time to return to daily activities, including weight-bearing, work, and jogging, also showed no statistical difference between groups. These outcomes, which directly reflect the functional impact of surgery on patients’ lives, are crucial in guiding surgical decision-making and patient counseling. The absence of an adverse effect from omitting the tourniquet in these parameters suggests that surgeons can safely adjust their intraoperative approach based on individual patient factors and intraoperative findings rather than routine use. These findings are further supported by a recent randomized controlled trial by D’Ambrosi et al., which demonstrated that tourniquet use during arthroscopic anterior cruciate ligament reconstruction did not enhance intraoperative visualization or reduce surgical time but was associated with significantly greater postoperative pain compared to procedures performed without a tourniquet, reinforcing the notion that avoiding its use may benefit early patient recovery without compromising surgical quality [38].

One of the major concerns associated with tourniquet use is ischemia–reperfusion injury, which may result in muscle damage, particularly in longer procedures. CPK levels, measured in the study by Tsarouhas et al., serve as an established biomarker of skeletal muscle injury. Across all postoperative time points (24 h, 8 days, and 15 days), no significant differences in CPK levels were observed between tourniquet and non-tourniquet groups, suggesting that tourniquet durations used in arthroscopic meniscectomy are likely too short to induce clinically significant muscle damage [39]. Moreover, systemic factors such as vitamin D deficiency have been shown to influence postoperative recovery and neuromuscular function, and should be considered in future investigations [40]. Nevertheless, it is important to interpret this result with caution. While serum markers may remain within normal limits, subclinical neuromuscular dysfunction, particularly in elderly or comorbid patients, might still occur and warrants further investigation in future studies using more sensitive neuromuscular assessment tools.

The type of anesthesia may also influence both intraoperative bleeding and patient outcomes. Recent studies concluded that neuraxial anesthesia, compared with general anesthesia resulted in a lower estimated blood loss [41,42,43,44,45]. Across the studies reviewed, a variety of anesthetic techniques were used, including general anesthesia, spinal or epidural block, and combined femoral–sciatic nerve block. Despite this heterogeneity, no consistent pattern emerged linking anesthetic technique with surgical efficiency or recovery metrics. However, the study by Gupta et al. notably combined general anesthesia with portal-site lidocaine infiltration and intra-articular adrenaline in one group, an approach that, when the tourniquet was omitted, still provided satisfactory operative conditions. This supports the growing evidence that pharmacologic strategies can successfully replace tourniquet use in many minimally invasive procedures. Given the small and heterogeneous evidence base, this review is primarily descriptive in nature. While it highlights trends in intraoperative and postoperative outcomes, it cannot provide definitive analytic conclusions.

### 4.1. Clinical Implications

From a practical standpoint, the findings of this review support a selective approach to tourniquet use in arthroscopic meniscectomy. Routine use may not be necessary, especially when alternative methods such as intra-articular adrenaline are employed [46]. Tourniquets may still be valuable as a backup tool in complex cases or in settings where bleeding control is insufficient. Importantly, avoiding unnecessary tourniquet use may reduce potential risks without compromising surgical quality or patient outcomes. Recent evidence has shown that although tourniquet use may improve intraoperative visualization during knee arthroscopy, it does not appear to confer significant benefits in long-term clinical outcomes and may be associated with increased postoperative discomfort, thereby supporting a more selective and individualized approach to its use [47,48,49,50].

### 4.2. Limitations and Research Gaps

This review provides important clinical insights, but it has several limitations. First, only three studies met the eligibility criteria, resulting in a small patient sample (243 patients). This reduces the possibility to detect rare complications or subtle differences. Second, there is no standardization in how the studies report their outcomes, particularly for subjective measures like visibility and surgeon satisfaction, introducing heterogeneity and potential bias. The implication of this was that our study had to include narrative representation of data at times. Future directions of research should include the development of a standardized and validated intraoperative visualization metric. Third, the included studies did not evaluate long-term results, such as continuous muscle weakness, deep vein thrombosis, osteoarthritic progression or functional limitations lasting longer than the immediate postoperative period.

Worth mentioning, both traumatic and degenerative tears were studied, with the former occurring in younger patients and the latter in older individuals. Degenerative tears are often associated with early osteoarthritic changes. This might suggest that age and osteoarthritis could be very important factors to consider in deciding whether a tourniquet is necessary or whether less invasive approaches would be just as effective [51].

Another limitation is that tourniquet pressures, application times, and surgical techniques showed variability, and this was not consistently covered across all studies. This inconsistency could limit the generalizability of the findings. None of the three studies explicitly defined surgical time. However, it should be noted that one trial equated surgical time with tourniquet inflation time, which may not be accurate since inflation and deflation do not always coincide with incision and closure [52]. This limits the reliability of operative time as a surrogate for true tourniquet duration. Finally, although randomized, none of the studies included used blinding of surgeons or outcome assessors, which might introduce some performance or detection bias.

### 4.3. Future Research Directions

Future investigations should prioritize the standardization of tourniquet protocols including clinical and radiographic follow up to capture residual muscle weakness, osteoarthritic progression and comprehensive patient reported outcomes on pain, activity, and quality of life. Rigorous thromboembolic surveillance, including routine duplex ultrasonography and other sensitive imaging, is needed to clarify whether tourniquet use alters the incidence of symptomatic or silent deep vein thrombosis after knee arthroscopy. Objective, validated metrics, potentially supported by digital imaging analytics, should be developed to quantify intraoperative visualization and bleeding control, thereby reducing subjective bias in future trials. Outcome data should be stratified by patient specific variables such as age, BMI, comorbidities, coagulation profile, and baseline muscle status to identify subgroups that derive benefit or incur harm from tourniquet application. Comparative studies must evaluate the effectiveness of mechanical limb occlusion versus pharmacologic vasoconstriction with agents such as intra articular adrenaline or tranexamic acid to establish optimal bleeding control strategies. Finally, comprehensive cost effectiveness analyses should weigh operative time, disposables, complications, and rehabilitation expenses to determine whether a selective tourniquet strategy offers economic as well as clinical advantages.

## 5. Conclusions

This review suggests that the use of tourniquets as a matter of routine in arthroscopic partial meniscectomy offers only slight intraoperative advantages, in terms of reduced operative time and improved visualization, especially when no pharmacological aids are used. Parameters such as pain intensity, early joint mobility, and overall recovery showed no meaningful differences between patients treated with or without a tourniquet. The current evidence, derived from only three heterogeneous RCTs with some risk of bias, suggests that routine tourniquet use in arthroscopic partial meniscectomy provides limited intraoperative benefits without improving postoperative outcomes, so the decision to use a tourniquet should be made on a case-by-case basis. Insufficient documentation of some aspects, such as tourniquet time, can severely hinder generalizability and no conclusions regarding long-term safety can be drawn. Therefore, these findings should be interpreted with caution and regarded as preliminary guidance rather than definitive recommendations. Further high-quality, large-scale RCTs are needed to create uniform clinical guidelines and to better understand the long-term effects of tourniquet use.

## Figures and Tables

**Table 1:** PICO search strategy.

P	Population	Adolescent and adult patients who underwent arthroscopic meniscectomy
I	Intervention	Tourniquet-assisted surgery during arthroscopic meniscectomy
C	Comparison	Non-tourniquet-assisted surgery
O	Outcomes	Operative time, blood loss and need for transfusion, postoperative drainage, intraoperative joint visibility, muscle strength levels, conversion rate to tourniquet, risk of short-term and long-term complications, patient-reported outcomes, intra-muscular enzymes, length of hospital stay

**Table 2:** Classification and Measurement of Primary and Secondary Outcomes.

Category	Outcome	Measurement	Unit
Primary	Operative time	From surgical incision to wound closure	minutes
Primary	Range of motion (ROM)	Knee flexion-extension at 1 and 2 weeks postoperatively	degrees
Primary	Postoperative pain	VAS at 1 and 2 weeks postoperatively	Visual Analogue Scale (0–10)
Primary	Time to return to function	Days to full weight-bearing, return to light work, and return to jogging	days
Primary	Knee effusion	Assessed at 1 week postoperatively	cm
Primary	Serum creatine phosphokinase (CPK) levels	Measured preoperatively, at 24 h,1 week, 2 weeks postoperatively	U/L
Secondary	Intraoperative visibility and surgeon satisfaction	Subjective scores based on operative field clarity	subjective score
Secondary	Volume of irrigation fluid used	Measured intraoperatively	mL
Secondary	Conversion to tourniquet use	Number of tourniquet-free cases requiring intraoperative conversion	count

**Table 3:** Study characteristics.

Study		Gupta (2021) [3]	Tsarouhas (2012) [30]	Junior (2010) [2]
Design		RCT	RCT	RCT
LoE		IB	IB	IB
RoB		9/10	8/10	6/10
Number of Patient	Total	60 *	80	103
	T group	20 ^(a)^	40	52
	NT group	20 ^(b)^	40 ^(c)^	51 ^(c)^
	NT2 group	20 ^(d)^	N/A	N/A
Age (years)	All	16–55	N/A	15–81
	T	32	34.8 ± 8.3	49.26 (20–81)
	NT	34	31.7 ± 8.9	49.17 (15–80)
	NT2	38	N/A	N/A
Female/Male		26/34	12/50	41/62
Type of meniscal tear		Traumatic	Traumatic	Traumatic, degenerative
Tourniquet pressure		100–150 mmHg higher than the systolic pressure, after elevation of the prepared limb for 3 min	320 mmHg after exsanguination of the limb by elevating it for 3 min	350 mmHg

Data presented as mean ± standard deviation (min–max) for numerical variables; Abbreviations: LoE, Level of Evidence; N/A, not applicable; NT, non-tourniquet; RCT, randomized controlled trial; T, tourniquet. Note: *, portal sites were injected locally with 2% lidocaine in all patients as per protocol; ^(a)^, portal site injections of 2% lidocaine along with tourniquet application; ^(b)^, portal site injections of 2% lidocaine only; ^(c)^, no local anesthesia; ^(d)^, portal site lidocaine injections were administered with 1:200,000 epinephrine.

**Table 4:** Measured outcomes and findings from included studies.

Study		Gupta (2021) [3]			Tsarouhas (2012) [30]			Junior (2010) [2]		
Group	T	NT	NT2	*p* Value	T	NT	*p* Value	T	NT	
No. of Patients	20 ^(a)^	20 ^(b)^	20 ^(c)^		40	40 ^(d)^		52	51 ^(d)^	*p* Value
Operative time (min)	19.25	26.25	20.75	0.001	27.5 ± 12.5	31.2 ± 12.1	0.83	21.29 (8–60)	21.71 (8–45)	0.8528
ROM, 1 week (°)					107.5 ± 17.8 (98–130)	105 ± 26.5 (95–132)	0.91			
ROM, 2 weeks (°)					142 ± 7.5 (130–148)	139.3 ± 11.4 (124–147)	0.96			
Difference in ROM between the knees (°)								8.36	8.70	0.8829
Pain, 1 week (VAS)					2.07 ± 1.9 (0–6)	1.25 ± 1.4 (0–5)	0.22			
Pain, 2 weeks (VAS)					0.7 ± 0.6 (0–2)	0.6 ± 1.26 (0–2)	0.43			
Effusion—Difference in perimeter between the knees, 1 week (cm)								0.269	0.279	0.9669
CPK levels, pre-op (U/L)					143.8 ± 29.20 (59–203)	171.2 ± 72.1 (52–290)	0.3			
CPK levels, 24 h (U/L)					100.9 ± 49.6 (41–211)	129.6 ± 45.2 (79–215)				
CPK levels, 8 days (U/L)					79 ± 24.3 (51–139)	163.5 ± 55.8 (69–211)	0.093			
CPK levels, 15 days (U/L)					61.3 ± 12.5 (46–89)	134.7 ± 28.9 (63–165)	0.079			
Surgeon satisfaction (S-VAS)	1.05	2.90	1.35	0.002						
Amount of fluid required (mL)	1895	2530	2105	0.001						
Conversion to tourniquet (count)	N/A	2	0	0.126						
Return to full weight bearing without crutches (days)					13.4 ± 3.35 (8–18)	13.2 ± 4.7 (8–21)	0.9			
Return to light work (days)					23.2 ± 5.3 (16–31)	24.6 ± 6.1 (15–36)	0.34			
Return to jogging (days)					35.7 ± 6.2 (24–48)	37.8 ± 7.3 (26–51)	0.23			

Data presented as mean ± standard deviation (min–max) for numerical variables. Abbreviations: N/A, not applicable; NT, non-tourniquet; S-VAS, surgeon visual analogue scale; T, tourniquet; VAS, visual analogue scale. Note: ^(a)^ portal site injections of 2% lidocaine along with tourniquet application; ^(b)^ portal site injections of 2%lidocaine only; ^(c)^ portal site lidocaine injections were administered with 1:200,000 epinephrine; ^(d)^ no local anesthesia.

## Data Availability

All data from this study are available upon reasonable request to the corresponding author.

## Supplementary Materials

The following supporting information can be downloaded at: https://www.mdpi.com/article/10.3390/jcm15052086/s1, S1: PRISMA 2020 for Abstract Checklist; S2: PRISMA 2020 Checklist.

## Abbreviations

The following abbreviations are used in this manuscript:

ACL	Anterior cruciate ligament
CCTs	Controlled (non-randomized) clinical trials
LoE	Levels of evidence
PRISMA	Preferred reporting items for systematic reviews and meta-analyses
PROSPERO	International prospective register of systematic reviews
ROM	Range of motion
RCTs	Randomized controlled trials
TKA	Total knee arthroplasty
VAS	Visual analogue scale

## Appendix A

Search strategy

**Filter:**

- Year, last 20 Years

**Databases:**

- Medline (via PubMed)

(“Tourniquets”[Mesh] OR Tourniquet* OR “no Tourniquet*”) AND (“Meniscectomy”[Mesh] OR Arthroscop* OR “Meniscectom*” OR “arthroscopic meniscectom*”)

221 results

- **Web of Science**

(ALL = (Tourniquet)) AND ((ALL = (Meniscectomy)) OR ALL = (arthroscopic meniscectomy))

29 results

- **Google Scholar**

(“Tourniquets”[Mesh] OR Tourniquet* OR “no Tourniquet*”) AND (“Meniscectomy”[Mesh] OR Arthroscop* OR “Meniscectom*” OR “arthroscopic meniscectom*”)

55 results

- **Scopus**

(Tourniquet AND (Meniscectomy OR Arthroscopic Meniscectomy))

35 results

- **Cochrane Library**

**ID**	**Search**	**Hits**
#1	Arthroscopic Meniscectomy	369
#2	Meniscectomy	585
#3	Tourniquet	2687
#4	(#1 OR #2) AND #3	22

## Appendix B

Title ICC

**Table A1:** Intraclass Correlation Coefficient.

	Intraclass Correlation ^b^	95% Confidence Interval		F Test with True Value 0			
		Lower Bound	Upper Bound	Value	df1	df2	Sig
Single Measures	0.440 ^a^	0.340	0.529	2.633	291	291	0.000
Average Measures	0.611 ^c^	0.508	0.692	2.633	291	291	0.000

Two-way mixed effects model where people effects are random and measures effects are fixed. ^a^. The estimator is the same, whether the interaction effect is present or not. ^b^. Type A intraclass correlation coefficients using an absolute agreement definition. ^c^. This estimate is computed assuming the interaction effect is absent, because it is not estimable otherwise.

Abstract ICC

**Table A2:** Intraclass Correlation Coefficient.

	Intraclass Correlation ^b^	95% Confidence Interval		F Test with True Value 0			
		Lower Bound	Upper Bound	Value	df1	df2	Sig
Single Measures	0.740 ^a^	0.587	0.842	6.626	51	51	0.000
Average Measures	0.851 ^c^	0.740	0.914	6.626	51	51	0.000

Two-way mixed effects model where people effects are random and measures effects are fixed. ^a^. The estimator is the same, whether the interaction effect is present or not. ^b^. Type A intraclass correlation coefficients using an absolute agreement definition. ^c^. This estimate is computed assuming the interaction effect is absent, because it is not estimable otherwise.

Full text ICC

**Table A3:** Intraclass Correlation Coefficient.

	Intraclass Correlation ^b^	95% Confidence Interval		F Test with True Value 0			
		Lower Bound	Upper Bound	Value	df1	df2	Sig
Single Measures	1.000 ^a^				5		
Average Measures	1.000 ^c^				5		

Two-way mixed effects model where people effects are random and measures effects are fixed. ^a^. The estimator is the same, whether the interaction effect is present or not. ^b^. Type A intraclass correlation coefficients using an absolute agreement definition. ^c^. This estimate is computed assuming the interaction effect is absent, because it is not estimable otherwise.
